# Development of a patient reported outcome measure for fatigue in motor neurone disease: the Neurological Fatigue Index (NFI-MND)

**DOI:** 10.1186/1477-7525-9-101

**Published:** 2011-11-22

**Authors:** Chris J Gibbons, Roger J Mills, Everard W Thornton, John Ealing, John D Mitchell, Pamela J Shaw, Kevin Talbot, A Tennant, Carolyn A Young

**Affiliations:** 1 Walton Centre for Neurology and Neurosurgery, Lower Lane, Liverpool, UK; 2 Department of Psychology, The University of Liverpool, Bedford Street South, Liverpool, UK; 3 Department of Neurology, Hope Hospital, Stott Lane, Greater Manchester, UK; 4 Royal Preston Hospital, Sharoe Green Lane, Preston, UK; 5 Sheffield Institute of Translational Neuroscience (SITraN), University of Sheffield, 385A Glossop Road Sheffield, UK; 6 Department of Clinical Neurology, John Radcliffe Hospital, Oxford, UK; 7 Academic Department of Rehabilitation Medicine, University of Leeds, Leeds, UK

## Abstract

**Background:**

The objective of this research was to develop a disease-specific measure for fatigue in patients with motor neurone disease (MND) by generating data that would fit the Rasch measurement model. Fatigue was defined as reversible motor weakness and whole-body tiredness that was predominantly brought on by muscular exertion and was partially relieved by rest.

**Methods:**

Qualitative interviews were undertaken to confirm the suitability of a previously identified set of 52 neurological fatigue items as relevant to patients with MND. Patients were recruited from five U.K. MND clinics. Questionnaires were administered during clinic or by post. A sub-sample of patients completed the questionnaire again after 2-4 weeks to assess test-retest validity. Exploratory factor analyses and Rasch analysis were conducted on the item set.

**Results:**

Qualitative interviews with ten MND patients confirmed the suitability of 52 previously identified neurological fatigue items as relevant to patients with MND. 298 patients consented to completing the initial questionnaire including this item set, with an additional 78 patients completing the questionnaire a second time after 4-6 weeks. Exploratory Factor Analysis identified five potential subscales that could be conceptualised as representing: 'Energy', 'Reversible muscular weakness' (shortened to 'Weakness'), 'Concentration', 'Effects of heat' and 'Rest'. Of the original five factors, two factors 'Energy' and 'Weakness' met the expectations of the Rasch model. A higher order fatigue summary scale, consisting of items from the 'Energy' and 'Weakness' subscales, was found to fit the Rasch model and have acceptable unidimensionality. The two scales and the higher order summary scale were shown to fulfil model expectations, including assumptions of unidimensionality, local independency and an absence of differential item functioning.

**Conclusions:**

The Neurological Fatigue Index for MND (NFI-MND) is a simple, easy-to-administer fatigue scale. It consists of an 8-item fatigue summary scale in addition to separate scales for measuring fatigue experienced as reversible muscular weakness and fatigue expressed as feelings of low energy and whole body tiredness. The underlying two factor structure supports the patient concept of fatigue derived from qualitative interviews in this population. All three scales were shown to be reliable and capable of interval level measurement.

## Introduction

Fatigue is one of the most commonly reported symptoms in motor neurone disease (MND) [1,2]. The etiology of this symptom is not yet fully understood and its progression and symptom salience varies between individuals. It has been shown to be associated with poor quality of life (QoL) [1], though there is some debate as to its precise relationship with concomitant disease factors, including depression [2].

Fatigue is an essentially subjective phenomenon; clinically, it remains undefined due to the overlap between the lay notion of tiredness and the clinically relevant symptom of fatigue [3]. In addition, fatigue may confound with loss of motivation or other symptoms. The symptom of fatigue extends beyond just muscular fatigability or weakness, it is distinct from depression and does not necessarily correlate with severity of disease [4]. Recent evidence supports the notion that fatigue in MND is an independent factor not directly associated with depression, dyspnoea or sleepiness [2].

The lack of research relating to fatigue in this population may be due in part to lack of tools available to accurately measure the experience of fatigue in MND. There are currently no MND-specific scales for measuring fatigue and it is long established that generic questionnaires may be insensitive to the unique experience of a patient with MND [5]. Similarly it has been demonstrated that the experience of fatigue may differ among neurological conditions [3]. In light of these considerations, there is a clear need to develop and validate a disease specific fatigue inventory for patients with MND. Without access to a valid tool for measuring and comparing levels of fatigue in this population, there is little hope for developing better treatment modalities that will allow this disabling symptom to become better managed.

The objective of this research is to develop a disease-specific measure for fatigue in patients with motor neurone disease (MND) by generating data that would fit the Rasch measurement model

## Methods

The Neurological Fatigue Scale for MND (NFI-MND) was developed in two stages: a confirmatory qualitative phase followed by a stage of formal psychometric assessment. Ethical permission was granted for both phases from relevant hospital committees in the U.K. (Sefton 05/Q0401/7 and Tayside 07/S1402/64), and local research governance committees at all participating sites.

Qualitative methodology was used to assess patient perception of fatigue in MND. A sample of 10 patients who had reported experiences of fatigue were interviewed at the time of their clinical visit. Participants all had a diagnosis of MND from a neurologist with expertise in MND. The interviews commenced with an open-ended question asking patients to describe their experience of fatigue. The interviews were then extended into a semi-structured format in which issues relating to fatigue derived from interviews with other samples of patients with neurological illness (including multiple sclerosis (MS), and stroke) were explored with the patients. In accordance with interpretative phenomenological analysis (IPA) guidelines [6] an *a priori *sample of ten patients was hypothesised to be sufficient to investigate the phenomenon of fatigue in patients with MND.

All patients who completed the qualitative interviews were then presented with the original pool of 52 items related to fatigue, developed initially for use in MS [7]. They were asked to comment on the relevance of the item set for MND and whether or not the items were understandable. The qualitative methodology is described in further detail elsewhere [8]. In addition, the MND qualitative data were compared to previously derived themes in MS for the emergence of new themes.

The psychometric and scaling properties of the proposed 52-item NFI-MND were then assessed among patients recruited from five regional MND care centres: The Walton Centre for Neurology and Neurosurgery in Liverpool, Preston Royal Hospital, Oxford John Radcliffe Hospital, Salford Hope Hospital and Sheffield Royal Hallamshire Hospital. Patients were eligible to enter the study irrespective of age, sex, and disease sub-type or disability status. Questionnaires were either handed out during a routine clinic appointment or sent to the patient's home, as part of a larger questionnaire pack sent alongside a newsletter describing the research activities of their local care centre. A subsample of patients completed The Modified Fatigue Impact Scale [9]. Two to four weeks after completing the first questionnaire patients were invited to complete a second questionnaire to assess test-retest reliability.

The Rasch measurement model was used to evaluate the scaling properties and construct validity of the 52-item draft questionnaire [10]. The Rasch model supplements the traditional psychometric assessments of reliability and construct validity by also evaluating the fundamental scaling properties of an instrument. The model operationalises the formal axioms of measurement (order, unidimensionality and additivety) allowing interval level data to be gained from questionnaires [11]. In the context of fatigue, the Rasch model simply states that the probability of a person affirming an item is a logistic function of the symptom severity the person experiences and the severity of the symptom measured by the question. For example if a person with a very low level of fatigue attempts a question that expresses a high level of fatigue, there is a high probability that they will not affirm the item. A detailed explanation and a more comprehensive review of Rasch methods may be found elsewhere [12].

To assess external validity, a visual analogue scale (VAS) of fatigue was included with the questionnaire pack. The question was marked on a 0-100 scale and prompted respondents to "Mark on the line, how severe you fatigue has been over the past 4 weeks". The VAS extremes were marked as 'Lively and alert' at the lower extreme and 'Absolutely no energy to do anything at all' at the upper.

### Analysis Procedure

An initial exploratory factor analysis (EFA) based on a polychoric correlation matrix was undertaken followed by an oblique Promax rotation. The objective at this stage is to avoid bringing to the Rasch analysis any serious multidimensionality. Thus an EFA is undertaken to give an indication of the dimensionality of the draft scale prior to more rigorous tests of unidimensionality within Rasch analysis [13]. Consequently a parsimonious solution is sought from the EFA, where a root mean square error of approximation (RMSEA) value below .10 is considered suitable [14].

### Fit to the Rasch model

Data are required to meet Rasch model expectations, and a number of fit statistics are used for this purpose. Fit is indicated by a non-significant summary chi-square statistic. Person and Item fit is also represented by residual mean values, where the summary fit standard deviation falls below 1.4, and individual person and item residuals fall within the range of ± 2.5.

### Local dependency

An assumption of the Rasch model is that items are locally independent, conditional upon the trait being measured (*i.e. *fatigue). This is identified by residual item correlations of +.3 and above. Where local dependency occurs items are too similar, and this artificially inflates reliability. This can be accommodated by summing the items together into one 'super' item, known as a testlet.

### Differential Item Functioning (DIF) [15]

Differential Item Functioning (DIF) occurs when different groups within the sample (*e.g. *males and females) respond in a different way to a certain question, given the same level of the underlying trait (*i.e. *fatigue). DIF occurs where there is difference in responses across groups. DIF would occur, for example, if men consistently give a higher score to an item than women, regardless of their level of fatigue. Analysis of variance (ANOVA, 5% alpha) is used to measure DIF. In the current study DIF was assessed for five factors: Test/Retest; Location (Liverpool, Oxford/Preston/Salford/Sheffield); Mode of Administration (clinic/delivered to home); Age (quartile split between participants) and Gender. Differential item functioning is used to examine contextual factors for invariance, preventing such factors being a source of confounding effect in the phenomenon being measured.

### Item Category Thresholds

The Rasch model also allows for a detailed analysis of the way in which response categories are understood by respondents. For example, in the case of a Likert style response, some respondents may have difficulty differentiating between categories, such as "Never" or "Very Rarely". In instances where there is too little discrimination between two response categories on an item, collapsing the categories into one response option can often improve scale fit to the Rasch model.

### Person Separation Index

This indicates the extent to which items distinguish between distinct levels of functioning (where .7 is considered a minimal value for group use; .85 for individual patient use).

### Unidimensionality

Finally, a series of independent t-tests are employed to assess the final scale for unidimensionality. Two estimates are derived from items forming high positive and high negative loadings on the first principal component of the residuals. These are compared and individual t-tests calculated. The number of significant t-tests outside the ± 1.96 range indicates whether the scale is unidimensional or not. Generally, less than 5% of significant t-tests are considered to be unidimensional (or the lower bound of the binomial confidence interval overlaps 5%) [12].

### Scale item reduction

Items are removed where necessary one at a time. Once an item is removed from a scale the resultant scale is reassessed for fit, dimensionality, local dependency and DIF. This iterative process is repeated until an acceptable solution is found for the scale.

The unrestricted 'partial credit' Rasch polytomous model was used with conditional pair-wise parameter estimation [16]. Rasch Unidimensional Measurement Model 2020 (RUMM2020) software (Version 4.1, Build 194) was used for the Rasch analyses presented in this study [17].

## Results

### Qualitative item validation

All themes in the item set were confirmed as being relevant to MND patients. All ten patients agreed that the areas covered by the 52 items were sufficient to capture all of their own personal experiences of fatigue, and no additional themes emerged from the interviews. A summary of the item framework, features, wording and supporting quotes taken from the qualitative investigation are given in Table 1. All patients filled out the draft scale and commented that all items were easy to understand and were relevant to their experience.

**Table 1:** Comparison of Item framework, feature, wording and supporting quotes

Framework	Feature	Example item	Supporting quote
Motor features	Can develop weakness	Sometimes, I lose my body strength	I have lost the ability to sustain [my strength]
Cognitive features	Concentrate on simple tasks	Sometimes, I have to concentrate on what are usually simple things	When reading a magazine or something and I'm getting tired then I find it more difficult to concentrate, it's just more difficult when I'm tired.
Motivation	Thought puts off doing	The thought of having to do something often puts me off doing it	[Because of] what's happened to get the fatigue you are less likely to want to do the same things anyway.
Tiredness	Tiredness	By the end of the day, I'm shattered	Just tiredness, all the time tiredness
Cadence	Carry over	If I've overdone things, I know about it the next day	I try not to do too much, because that'd knacker me up for the next day then
Precipitating/aggravating factors	Physical exertion induces weakness	I soon become weak after physical effort	[I get fatigued] when doing anything physical, it's surprising that the things I never used to regard as physical are now physical and causing fatigue.
Relieving factors	day rest restorative	Resting allows me to carry on	[I'd rest for] maybe three to four minutes, something like that. I make sure that it is long enough if it's any shorter then I'm back to square one; it feels like I am not recharged enough.
Severity	weak at rest	I can become weak even if I've not been doing anything	Now you just do very minimal things that you wouldn't consider as anything and you feel fatigued
Associated features	Unrefreshing nocturnal sleep	When I wake in the morning, I get the weariness feeling	I wake up in the morning, and get this weariness feeling

### Quantitative scale validation

#### Patients

For the main data collection, 278 questionnaires sent to the patient's homes were returned, and a further 20 were completed during a routine clinic appointment. In total 544 questionnaires were sent to patients (54.7% response). Completeness for the 52 items of the MND NFI was 98.53%. To assess test-retest validity, 78 patients completed the pack for a second time after a period of 4-6 weeks. One hundred and eighty five participants completed the MFIS. The average age of participants was 62.1 ± 11 years. In total, 186 respondents (62.1%) were male. Contemporaneous functional status information for 141 patients (25 at retest) was collected from clinical notes no more than 1 month prior to or following completion of the questionnaire (Amyotrophic Lateral Sclerosis Functional Rating Scale Revised - ALSFRS-R [15]). Summary demographic information and questionnaire response by centre is displayed in Table 2.

**Table 2:** Demographics and questionnaire response per centre.

Demographics		Test	Retest
	Age (years)	62.1 ± 11	63.2 ± 10.5
	Sex	62.4% male	64.9% male
	Returned	298	82
	Clinic	20	0
	Home	278	82
	ALSFRS-R	32.72 ± 8.27*	31.92 ± 9.89**
	Disease duration (years)	2.69 ± 3.54	2.73 ± 2.88

Centre	Liverpool	110	36
	Sheffield	38	5
	Oxford	39	9
	Manchester	76	13
	Preston	35	15

* N = 141 ** N = 25

#### Exploratory factor analysis

The data from the 298 respondents were subjected to an exploratory factor analysis (EFA). This indicated an acceptable 5-factor solution with an RMSEA of .10. The factors were thematically conceptualised to reflect 'Lack of energy' (15 items), 'Weakness' (9 items), 'Effects of Heat' (4 items), 'Concentration' (4 items) and 'Rest' (4 items).

#### Rasch Analysis

##### Rest and Concentration Subscales

Only 4 items loaded exclusively onto each of the 'Rest' and 'Concentration' subscales. Due to the small number of items loading on each factor, after dealing with misfitting items, neither subscale could be reconciled to meet Rasch model demands.

##### Effects of Heat Subscale

The 'Effects of Heat' component was omitted from the Rasch analysis of the final scale based on qualitative evidence that for patients with MND, that extreme temperature was an effect modifier (*i.e. *made fatigue better or worse) rather than directly related to fatigue. In addition only 4 items loaded to this subscale.

Data for the 'Energy' and 'Weakness' domains were then fitted to the Rasch measurement model. An iterative process of item reduction involved identifying disordered thresholds, differential item functioning, item misfit, breaches of local dependency and multidimensionality. A summary of findings related to the analysis of both domains, and the final summary scale, are given in Table 3.

**Table 3:** Summary Analysis figures for Rasch analyses of the NFI:MND

		Item Residual		Person Residual		Chi Square		PSI	Unidimensional t-test (CI%)	% extreme scores
				
Analysis Name	# of items	Mean	± SD	Mean	± SD	Value	p			
1. Energy Initial	15	-0.34	2.21	-0.46	1.61	91.94	< 0.0001	0.93	9.34% (7-12)	0.80%
2. Energy Final	6	0.25	1.08	0.46	1.13	12.63	0.81	0.88	3.86%	4.40%
3. Weakness Initial	9	0.41	2.74	0.27	1.30	106.5	< 0.0001	0.86	2.79%	3.70%
4. Weakness Final	7	-0.08	1.36	-0.41	1.15	29.24	0.11	0.86	4.24%	5.10%
5. Summary Initial	13	-0.72	1.56	-0.42	1.38	70.22	0.04	0.92	4.44%	1.70%
6. Summary Final	8	0.11	1.25	-0.4	1.20	32.93	0.42	0.86	5.15% (3-8)	2.40%
***Ideal Values***		***0***	***< 1.4***	***0***	***< 1.4***		***> 0.05^a^***	***> 0.85***	***< 5% (CI < 0.05)***	

a = Bonferroni corrected

##### Energy Subscale

Initial fit of the 15 items to the Rasch model was poor, with person and item means exceeding the expected values. The item set displayed multidimensionality (see Table 3. Analysis 1). An iterative process led to scale reduction of 9 items. The resulting 'Energy' subscale showed good fit to model expectations, including unidimensionality, ordered category thresholds as well as an absence of both differential item functioning (DIF) and local dependency (see Table 3. Analysis 2). Principal component analysis revealed that 63.37% of the variance in fatigue was explained by the energy subscale. Individual item fit statistics for the Energy subscale are presented in Additional File 1.

##### Weakness Subscale

All thresholds were correctly ordered for the nine item scale. Two items: *'I have problems with my speech when I am tired' *and *'The cold makes my body very stiff' *displayed substantial misfit to the Rasch model and failed to meet scale expectations (Table 3, Analysis 3). Removal of the misfitting items improved fit of the scale, yielding strict unidimensionality, no DIF, and supported the local independence assumption (Table 3, Analysis 4). The weakness subscale accounted for 52.79% of the variance of fatigue. Individual item fit statistics for the Weakness subscale are presented in Additional File 1.

##### Summary Scale

All items from the 'Weakness' and 'Energy' subscales were then included as potential items for a summary fatigue scale (a higher-order factor). The 13 items showed reasonable fit to the Rasch model, though the standard deviation of the item fit residual was above the expected value. An iterative procedure reduced the summary scale to 8 items, producing a unidimensional scale with excellent fit to the Rasch model (Table 3, Analysis 6). Principal component analysis revealed that 52.09% of the variance in fatigue was explained by the summary scale. Individual item fit statistics for the summary scale are presented in Additional File 1.

##### Scale Targeting

**Figure 1:**
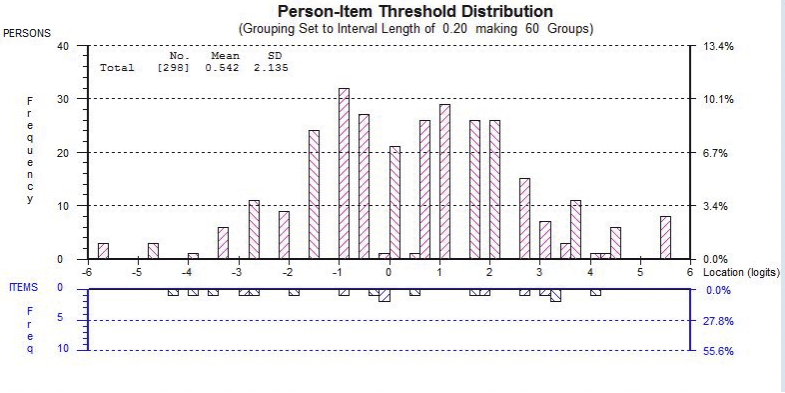
**Person-item threshold distribution for Energy subscale**.

The three final scales (Weakness, Energy and the Summary scale) showed acceptable person-item targeting (see Figure 1. for example) with extreme scores less than 5% in all cases. In Figure 1 person locations are shown above the *x-*axis and represent the amount of fatigue patients have, bars below the *x-*axis represent item threshold location (the amount of fatigue measured by the items). Good scale targeting is indicated by a good spread of item threshold locations that correspond to person locations above the *x*-axis. Person-item threshold distribution graphs for the Weakness and Summary scales are provided in Additional Files 2 and 3.

##### Test-Retest reliability

Retesting was performed between two and four weeks. The invariance of the scales over time was confirmed by the absence of DIF by time. Test-retest reliability was good, with correlation coefficients all above .65. There were no significant differences in the mean scores (median for Energy subscale) between time points (Paired Samples T-Test and Wilcoxon Signed Rank; p > 0.05 (see Table 4).

**Table 4:** Test-retest comparison for NFI:MND

Scale	T1-T2 Correlations	Mean Scores T1, T2	SD (q1-q3)	Mean difference	% of cases falling within 95% confidence interval
Summary	0.84*	14,14^a^	9.79 (7-13)	0.10	94.03%
Energy	0.67**	10,10^b^	13.09 (11-16)	0.15	89.66%
Weakness	0.86*	13,13^a^	17.42 (14-21)	0.10	92.54%

* Pearson's r

**Spearman's Rho

a Paired Samples T-Test; p > 0.0001

b Wilcoxon Signed Rank; p > 0.0001. Note median score used due to sample distribution

Key - SD = Standard deviation, q1 = Quartile One, q3 = Quartile Three

Bland and Altman [18] analysis was conducted to assess test-retest repeatability. Mean differences did not exceed 1 point on the 100 point scale, meaning they were clinically insignificant (see Table 4). For all three scales 89-95% of cases fell within the 95% confidence interval constructed for a normal distribution. Bland Altman plots for the three scales are available in Additional Files 4, 5 and 6.

##### Differential Item Functioning

No DIF was revealed for any of the five examined person factors for any of the scales, indicating the NFI-MND may be administered to patients in the U.K. regardless of age or gender, at a clinic appointment or at the patient's home via postal administration.

##### External construct validity

To assess external construct validity, raw scores on the NFI-MND were compared to those from a VAS measure of fatigue using Pearson's product-moment correlation. The summary, energy and weakness subscales correlated with VAS scores for fatigue to a magnitude of .60, .65 and .54 respectively. One hundred and eighty five respondents also completed the Modified Fatigue Impact Scale (MFIS) [9] at the same time as the MND-NFI. Pearson product moment correlations between the scales of the MNDNFI and the MFIS were strong (Energy r = .66, p < 0.0001; Weakness r = .71, p < 0.0001; Summary r = .75, p < 0.0001).

The relationship between the NFI-MND scales and the ALSFRS-R measure of functional status was explored using data collected from hospital notes for 141 of the study participants. Pearson's correlation values using raw score data reveal that functional status correlated mildly with the summary fatigue scale (r = -.18, *p *= 0.03) and the weakness subscale (r = -.23, *p *= 0.005), the energy subscale did not correlate significantly with functional ability (r = -.07, *p *= 0.41). In accordance with past research, these results suggest that there is no simple linear relationship between fatigue and functional status for patients with MND [2].

##### Raw score to interval scale conversion

Table 5 provides a simple chart for allowing conversion of raw scores taken from each of the three scales into interval level scores for use in arithmetic operations. These conversions will hold provided there is no missing data. Use in parametric analyses will also require appropriate distributional properties.

**Table 5:** Conversion table for raw to interval scores.

Raw Score	Summary Scale	Weakness Subscale	Energy Subscale
0	0.00	0.00	0.00
1	2.05	1.88	1.53
2	3.56	3.29	2.79
3	4.66	4.35	3.83
4	5.59	5.24	4.80
5	6.41	6.04	5.75
6	7.17	6.78	6.70
7	7.90	7.50	7.61
8	8.62	8.20	8.48
9	9.34	8.91	9.31
10	10.07	9.63	10.14
11	10.81	10.36	10.97
12	11.58	11.13	11.81
13	12.36	11.91	12.63
14	13.16	12.73	13.45
15	13.98	13.58	14.29
16	14.80	14.47	15.24
17	15.62	15.41	16.45
18	16.44	16.43	18.00
19	17.28	17.59	
20	18.17	19.08	
21	19.16	21.00	
22	20.32		
23	21.89		
24	24.00		

Table 5 may be used to translate raw total scores taken from the questionnaires into interval level scores suitable for use in mathematic operations. NB: Only valid with complete data.

## Discussion

The purpose of this study was to develop and validate a disease-specific instrument for measuring fatigue in patients with MND. Qualitative analysis confirmed the suitability of a previously identified 52-item neurological fatigue set. Rasch model expectations were met after correctly ordering the item set into salient factors and removing misfitting items.

As expected for this functionally limited population, the themes of the final scale were not heavily focussed around fatigue following strenuous exercise. Generic instruments, such as the Fatigue Severity Scale [19], include items assessing fatigue following levels of exertion that are simply not possible for patients in the later, disabling stages of MND. For example, the Multidimensional Fatigue Inventory [20] measures fatigue over 20 items split into 5 dimensions; General Fatigue, Physical Fatigue, Mental Fatigue, Reduced Motivation and Reduced Activity. Our qualitative findings suggest that patients with MND not only make minimal reference to activity determined fatigue in the classical sense (*i.e. *following exercises such as running) but report fewer experiences of mental fatigue than patients with other neurological disorders, such as multiple sclerosis [21].

There are some of limitations to the study. Whilst we endeavoured to obtain a representative sample, most patients were recruited initially either at a routine clinic appointment or where the patient was known to the clinical team to be interested in research. Selecting patients in this manner may have caused the sample to be skewed toward patients who were at early stages of the disease rather than those nearing the end stage of the disease, although ALSFRS-R scores suggested a wide spread of disability within our sample. Additionally, the number of ALSFRS-R responses restricts the power of correlations to detect changes below magnitudes of r = 0.2. However, other researchers [2] have found there to be no significant relationship between functional status and fatigue in patients with MND.

Scores for test-retest reliability for the Energy subscale were slightly below expected values. Test-retest reliability analyses were carried out between two to four weeks after the completion of the original questionnaire. The rapidly progressive nature of MND could mean that, for some patients, a large increase in this aspect of fatigue may occur within a four week period. The current study may have been improved by collecting test-retest data over a shorter time period, in order to minimise the effects of the rapid natural progression of the disease upon the results of test-retest reliability analyses.

Differential item functioning analyses in this study were limited by the small sample sizes in the clinic completion group, which contained only twenty patients. Small numbers are apparent in this group due to the difficulties of administering a suite of questionnaires, including a 52-item fatigue measure, in a short clinic appointment. Many patients expressed a preference to take the pack home to complete. The thirteen items of the MND-NFI are now more suitable for clinic administration and further work may usefully examine the validity of the MND-NFI for clinician administration, as well as patient self-complete.

An important caveat of disease specific outcome measures is their inability to provide comparisons between disorders [22] that may serve to foster a more complete understanding of fatigue and its mechanisms. However, the Rasch model is capable of addressing this problem and allowing for comparisons to be made across different disease groups, especially if the scales have been derived in such a manner as to share common items [23]. Further progress could be made using the initial 52 questionnaire items to form the basis of other Rasch validated disease-specific scales for neurological conditions such as stroke and post-polio syndrome; allowing for both disease-specific measurement and inter-disease comparison. To this end, the Neurological Fatigue Index for Multiple Sclerosis (NFI-MS) was derived from the same initial 52 item bank and separately validated specifically for use in multiple sclerosis [7]. The NFI-MS measures fatigue over four domains revealed to be salient to patients with MS; 'Physical', 'Cognitive', 'Relief by diurnal sleep or rest' and 'Abnormal nocturnal sleep and sleepiness', although the latter two scales were acknowledged to be only provisional, and may indicate adaptive processes, rather than aspects of fatigue itself. Further work is warranted to compare fatigue as experienced by patients with MND, MS and other neurological illnesses.

In the NFI-MND the simple duality of the 'Weakness' and 'Energy' subscales will also assist clinicians in assessing what patients mean when they describe feelings of fatigue. As such the NFI-MND fatigue scale may serve as a valuable tool for assessing the patient experience of fatigue and how this disabling symptom changes over time in clinical settings, clinical trials and in bio-psychosocial research studies. This is facilitated further by the transformation of the ordinal raw scores into interval level measurement.

Importantly, the MND-NFI is a brief measure, containing only 13 items, with only 8 items in the summary scale. Questionnaire length is an important concern for patients with MND, particularly when they are suffering from fatigue [22]. The brevity of the MND-NFI makes it appropriate for routine clinical application in this population, but the scale may also be used in clinical trials, whilst the full NFI-MND may lend itself to bio-psychosocial and biological studies.

Given that all three scales fit the Rasch model, the raw score from each scale is sufficient for identifying the ordinal level of fatigue, energy or weakness a patient exhibits. This ordinal score is convenient for 'everyday' use and will give a good indicator of the levels of fatigue displayed by the respondents. Whenever parametric statistics are required for the data, the ordinal-interval conversion can be employed, in the event there are no missing data.

## Conclusion

The NFI-MND is a brief, easy-to-administer fatigue scale for patients with MND. It consists of an 8-item fatigue summary scale in addition to separate scales for measuring fatigue as experienced as reversible muscular weakness and fatigue expressed as feelings of low energy and whole body tiredness. The underlying two factor structure supports the patient concept of fatigue derived from qualitative interviews in this population. All three scales were shown to be reliable and capable of interval level measurement and are suitable for use in clinics or research.

### Implications for practice and research

The summary scale for the MND-NFI is suitable for use in both a clinical and research settings. Given fit to the Rasch model, the raw score is sufficient for identifying the ordinal level of fatigue in patients by simply adding the scores from the questionnaire. Where parametric statistics are required, a nomogram is provided for ordinal-interval transformation. The NFI-MND is free for use in all public health and not-for-profit agencies, and can be obtained from the authors following a simple registration.

## Competing interests

The authors declare that they have no competing interests.

## Authors' contributions

CJG collected data, conducted analyses and is the primary author of this manuscript. EWT assisted in study design and authoring of the paper and is a co-grant holder. RJM provided expert review and assisted in study design and editing.

JE, JDM, PJS and KT facilitated data collection in the MND care centres they run. AT provided expert statistical advice regarding Rasch analysis. CAY assisted in study design, authoring, collection of data and editing and in the primary grant holder. All authors read and approved of the finalised version of this manuscript.

## Supplementary Material

Additional file 1 **Item Fit Statistics for NFI-MND**. Individual item fit statistics for the Energy, Weakness, and Summary scales. Click here for file

Additional file 2 **Person-Item Distribution for Weakness subscale**. Illustration of Person-Item threshold distribution for the Weakness subscale. Click here for file

Additional file 3 **Person-Item Distribution for Summary scale**. Illustration of Person-Item threshold distribution for the Summary scale. Click here for file

Additional file 4 **Bland-Altman plot for Energy subscale**. Bland-Altman plot showing agreement between test and retest scores for the Energy subscale. Click here for file

Additional file 5 **Bland-Altman plot for Weakness subscale**. Bland-Altman plot showing agreement between test and retest scores for the Weakness subscale. Click here for file

Additional file 6 **Bland-Altman plot for Summary scale**. Bland-Altman plot showing agreement between test and retest scores for the Summary scale. Click here for file
