# Supplementary figures and images for: Development of a patient reported outcome measure for fatigue in motor neurone disease: the Neurological Fatigue Index (NFI-MND)
Source: Health Qual Life Outcomes. 2011 Nov 22;9:101. doi: 10.1186/1477-7525-9-101 (PMC3282643; doi:10.1186/1477-7525-9-101)

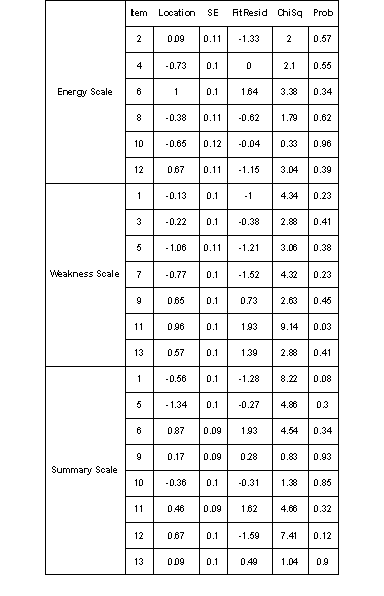

Supplement: Additional file 1 — Item Fit Statistics for NFI-MND. Individual item fit statistics for the Energy, Weakness, and Summary scales. [file 1477-7525-9-101-S1.BMP]

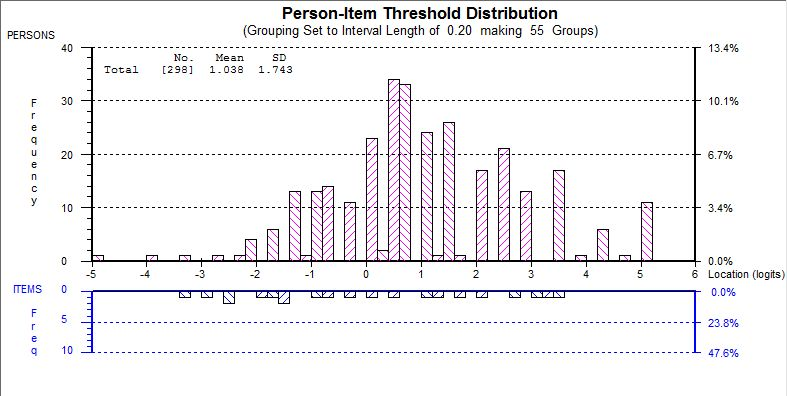

Supplement: Additional file 2 — Person-Item Distribution for Weakness subscale. Illustration of Person-Item threshold distribution for the Weakness subscale. [file 1477-7525-9-101-S2.BMP]

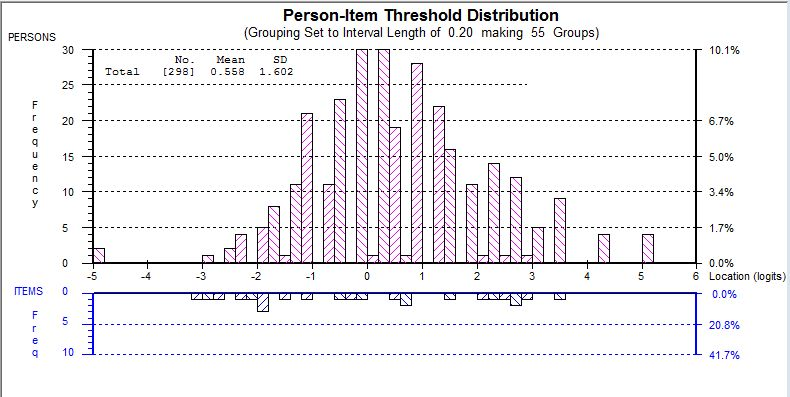

Supplement: Additional file 3 — Person-Item Distribution for Summary scale. Illustration of Person-Item threshold distribution for the Summary scale. [file 1477-7525-9-101-S3.BMP]

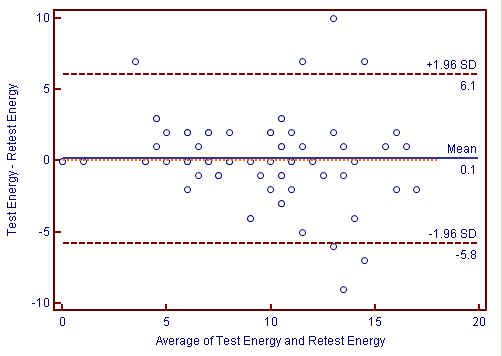

Supplement: Additional file 4 — Bland-Altman plot for Energy subscale. Bland-Altman plot showing agreement between test and retest scores for the Energy subscale. [file 1477-7525-9-101-S4.BMP]

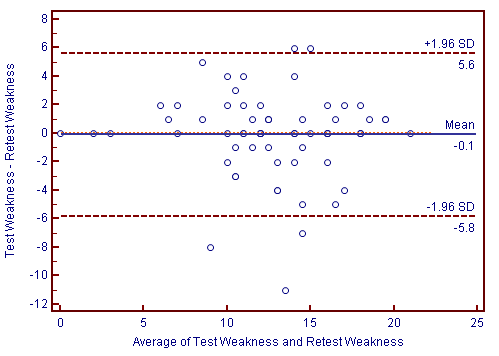

Supplement: Additional file 5 — Bland-Altman plot for Weakness subscale. Bland-Altman plot showing agreement between test and retest scores for the Weakness subscale. [file 1477-7525-9-101-S5.BMP]

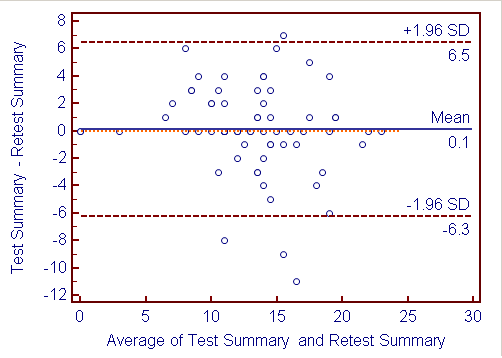

Supplement: Additional file 6 — Bland-Altman plot for Summary scale. Bland-Altman plot showing agreement between test and retest scores for the Summary scale. [file 1477-7525-9-101-S6.BMP]
